# Scanning of Adsorption Hysteresis *In Situ* with Small Angle X-Ray Scattering

**DOI:** 10.1371/journal.pone.0164636

**Published:** 2016-10-14

**Authors:** Athanasios Ch. Mitropoulos, Evangelos P. Favvas, Konstantinos L. Stefanopoulos, Etienne F. Vansant

**Affiliations:** 1 Hephaestus Laboratory, Department of Petroleum and Mechanical Engineering, Eastern Macedonia and Thrace Institute of Technology, Cavala, St. Lucas 654 04, Greece; 2 Membranes and Materials for Environmental Separations Laboratory, Institute of Nanoscience and Nanotechnology, National Centre for Scientific Research “Demokritos”, Ag. Paraskevi, 153 41, Athens, Greece; 3 Department of Chemistry, Laboratory of Adsorption and Catalysis, University of Antwerp, Universiteitsplein 1, B2610 Wilrijk, Belgium; Monash University, AUSTRALIA

## Abstract

Everett’s theorem-6 of the domain theory was examined by conducting adsorption *in situ* with small angle x-ray scattering (SAXS) supplemented by the contrast matching technique. The study focuses on the spectrum differences of a point to which the system arrives from different scanning paths. It is noted that according to this theorem at a common point the system has similar macroscopic properties. Furthermore it was examined the memory string of the system. We concluded that opposite to theorem-6: a) at a common point the system can reach in a finite (not an infinite) number of ways, b) a correction for the thickness of the adsorbed film prior to capillary condensation is necessary, and c) the scattering curves although at high-Q values coincide, at low-Q values are different indicating different microscopic states. That is, at a common point the system holds different metastable states sustained by hysteresis effects. These metastable states are the ones which highlight the way of a system back to a return point memory (RPM). Entering the hysteresis loop from different RPMs different histories are implanted to the paths toward the common point. Although in general the memory points refer to relaxation phenomena, they also constitute a characteristic feature of capillary condensation. Analogies of the no-passing rule and the adiabaticity assumption in the frame of adsorption hysteresis are discussed.

## Introduction

In the last ten years considerable effort has been made on studying capillary condensation/evaporation phenomena in mesopore systems. To this end, new materials with a well-defined pore geometry together with advanced computational modelling and simulation techniques have been employed [1–13]. According to IUPAC [14] mesopores are intermediate or transitional pores, between macro- and micropores, with sizes ranging from 500 down to 20 Å. Mesoporous materials are mostly known to exhibit a type IV adsorption isotherm with a hysteresis loop [15]. However adsorption [16–21] is only an example among several other physical processes (e.g. magnetism [22], solid transitions [23], contact angle [24], etc) that exhibit hysteresis phenomena. Extension of hysteresis to other fields of science and even social sciences [25] has also been made. When a system shows a hysteretic behaviour the usual two experimental variables (i.e. the independent and the dependent one) are not sufficient to fully describe the state of the system; one more (internal) variable, related to the history of the system, is required [26].

**Fig 1:**
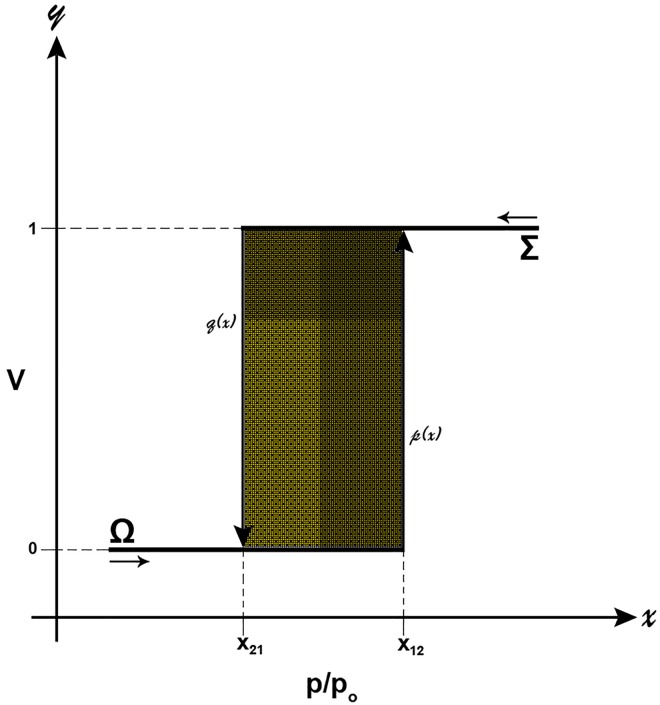
Preisach model of hysteresis (see text for details).

In order to demonstrate this, let us examine the behaviour of a single cylindrical pore (domain) of radius R_c_ in the Preisach model [27] (Fig 1). For this pore we have assumed perfect wetting and also negligible thickness of the adsorbed or remaining film prior to capillary condensation or after the capillary evaporation. When the pore is empty (state Ω) the amount adsorbed V_Ω_ = 0, whereas when filled (state Σ) V_Σ_ = 1. According to Cohan-Kelvin equation [28] this pore will be filled at a relative pressure (p/p_o_)_ads_ or x_12_ = e^-K/Rc^ and will be empty at (p/p_o_)_des_ or x_21_ = e^-2K/Rc^; where K = γV_L_/RT, γ and V_L_ are the surface tension and the molar volume of the liquid adsorbate, R is the gas constant and T is the absolute isothermal temperature. As always x_12_>x_21_, then for any x≤x_21_ the pore is empty and for any x≥x_12_ the pore is filled. However, for values of x between x_12_ and x_21_ it is not clear the state of the pore unless the history of the system process is known. If x before enters the range x_12_ to x_21_ has values x<x_12_ then V_Ω_ = 0 and if it has values x>x_21_ then V_Σ_ = 1.

Now, by denoting the required internal variable as ξ; where ξ is the fraction of pores in state Σ and since we have only one pore: a) when x enters the range x_21_→x_12_ (i.e. from Ω: values less than x<x_21_<x_12_) the pore is empty or ξ = 0 and b) when x enters the range x_12_→x_21_ (i.e. from Σ: values greater than x>x_12_>x_21_) the pore is filled or ξ = 1. In summary:

$$V\begin{array}{l} \Sigma \\ \Omega \end{array}\}=\left\{ \begin{array}{l} 1\;if\;x\geq x_{12}\\ 1\;if\;x_{21}<x<x_{12}\;and\;\xi =1\\ 0\;if\;x\leq x_{21}\\ 0\;if\;x_{21}<x<x_{12}\;and\;\xi =0 \end{array}\right.$$ (1)

It is noted however that the exact nature of ξ depends on the problem considered. For instance a thermodynamic property J of many domains n_o_ is given by:

$$\frac{\text{J}}{{\text{n}}_{\text{o}}}={\text{j}}_{\Sigma }\xi +{\text{j}}_{\Omega }(1-\xi )$$ (2)

where j’s are the molar contributions to J. Hysteresis phenomena are also characterized by the existence of scanning curves which are also stable and reproducible. The adsorption branch of the adsorption isotherm constitutes the ascending boundary p(x) and the desorption branch of the adsorption isotherm the descending boundary q(x). A journey within the hysteresis loop from a starting point on the ascending boundary results to a primary descending curve whereas from the descending boundary to a primary ascending curve and so on secondary and tertiary curves are defined.

Scanning curves can provide detailed information on the pore network as well as an insight into the nature of the hysteresis loop. A first approximation to this end is to treat the pore system as a set of domains independently acting. The idea of domains was first introduced by Ewing [29] in his classical theory of magnetic hysteresis and the independent-pore model is originated from Preisach [30] studies on the same topic. According to Everett [31–34] approach each domain is characterized by three variables, V(x_12_, x_21_) and a plot of this function will produce a 3-D surface. By flattening the surface to a 2-D representation a domain complexion diagram is obtained. Other versions of this diagram are the Preisach [30,35], Enderby [36,37], and Neel [38,39] diagrams (see for example in[40–45]). The latter is the simplest one where an isosceles right triangle gives the corresponding representation. The theory comprises 7 theorems whose validity has been questioned in the case of non-independent domains.

Enderby [36,37] extended domain theory to deal with more independent variables and also to take into account nearest neighbour interactions in a one-dimensional array of domains. Coasne et al. [44] have also extended the theory in order to account for the adsorbed film. Grosman and Ortega [45] discussed the effect of the adsorbed film by testing whether or not SBA-15 behaves as an assembly of independent domains. Rojas et al. [46] revisited Everett’s theorems by comparing independent and non-independent domain models in the frame of the dual-site bond model. They concluded that theorems 1–4 are of limited qualitative validity for non-independent pore domains whereas theorems 5–7 are valid in both independent and non-independent structures.

Lilly et al. [47] examined the hysteretic behaviour of helium in a porous material. They demonstrated memory effects but they also found deviations from the predictions of the domain theory which were attributed to avalanche events that take place within the porous system under investigation. On the memory effect, coined as return-point memory (RPM), Lilly and Hallock [48] concluded that it is a property of capillary condensation systems and as a matter of fact it is a robust one that does not need the assumption of the independent pores and is not limited to the boundary curves only; it extends to sub-cycles within cycles and so on. In other words the system can remember an entire hierarchy of turning points in its past external field. The memory effect can be explained by using Middleton’s [49] no-passing rule with the adiabaticity assumption. Sethna et al. [50] have discussed this case in conjunction with Preisach models [51–53].

In this article we have examined theorem-6 by conducting adsorption *in situ* with small angle x-ray scattering (SAXS) measurements. Our study focuses on what kind of differences a point to which the system arrives from different scanning paths can possibly shows. It is noted that according to this theorem at a common point the system has definite static macroscopic properties. Furthermore we speculate on the memory string of the system; i.e. what kind of memory [54,55] guides the system to take the correct path on departing from that common point.

## Experimental Procedures

In this study, we present *in situ* measurements on the hysteresis of dibromomethane (CH_2_Br_2_) onto Vycor porous glass using SAXS. Dibromomethane is able to ‘contrast match’ with amorphous silica; in this way, when a set of glass pores is filled with condensed CH_2_Br_2_ liquid, it will cease to act as scatterer and only the remaining empty pores will produce a measurable intensity. It should be noted, however, that the sample cell which facilitates this adsorption process in conjunction with SAXS measurements may introduce an error in the temperature (held at 20°C), and consequently in the relative pressure, of the order of ±0.2°C and ±0.01, respectively.

SAXS measurements were performed on a JJ X-ray system (Denmark) connected to a sealed tube CuKα x-ray generator (λ = 1.54 Å). The sample-to-detector distance and the beam centre were precisely determined by calibration with the Ag–behenate standard (d_001_ = 58.38 Å). Scattering data were corrected for dark current and empty tube scattering. The Q-range was varied from 0.004 to 0.1 Å^-1^; where Q = 4πsinθ/λ is the scattering vector, λ and 2θ are the wavelength and the scattering angle respectively.

Nitrogen adsorption measurements at -196°C (77 K) were performed using an Autosorb-1 static volumetric system (Quantachrome Instruments). Dibromomethane adsorption-desorption isotherms were conducted gravimetrically at 20°C by means of an Intelligent Gravimetric Analyser (IGA, Hiden Isochema). In both adsorption experiments the samples where outgassed overnight at 200°C under high vacuum (<10^−6^ mbar).

The Brunauer-Emmett-Teller (BET) area is found to be 80 m^2^/g for CH_2_Br_2_, the saturation (Gurvitsh) pore volume V_p_ = 209 mm^3^/g, and the porosity 31%. The Porod analysis gives a higher surface area for CH_2_Br_2_ (108 m^2^/g). Further, the BET area for N_2_ is 135 m^2^/g. All the information utilized by the dibromomethane adsorption in conjunction with SAXS measurements along the isotherm is discussed in detail in [4]. Since the SAXS experiment carried out in situ with CH_2_Br_2_ adsorption the relative intensities will reflect only the changes associated with the adsorption process. To this end, the interrelation between adsorption and scattering may be obtained by plotting g_Q_, which is the area under the scattering curve at a given p/p_0_ = x divided by the area under the scattering curve at p/p_0_ = 0.

$$g_{Q}=1-\frac{\int {\left[I(Q)\right]}_{x}dQ}{\int {\left[I(Q)\right]}_{0}dQ}$$ (3)

**Fig 2:**
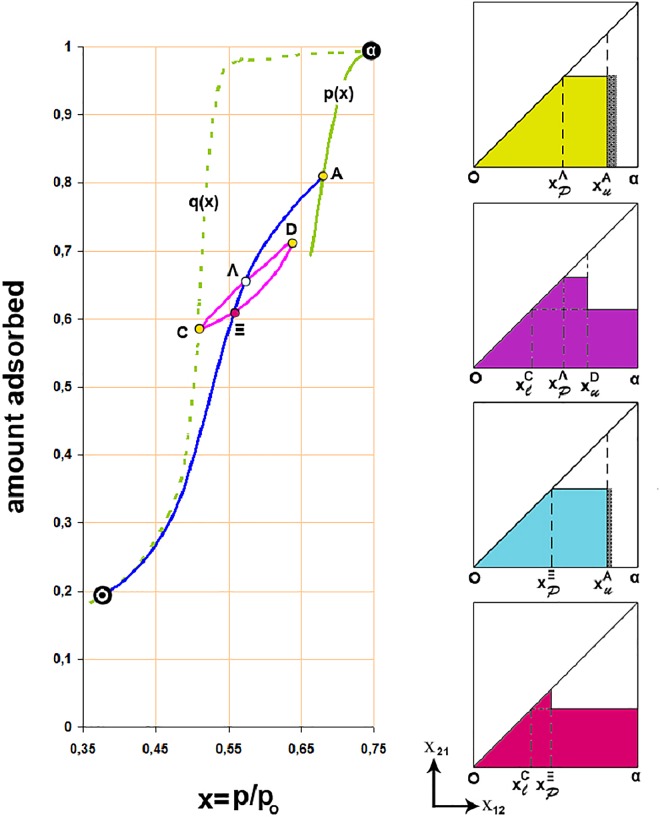
Effect of the adsorbed film on scanning the hysteresis loop of CH_2_Br_2_/Vycor adsorption isotherm according to theorem-6 of the domain theory. Points Λ and Ξ are approached from different routes; ΑΛΞΟ is a primary descending curve originated from the ascending boundary p(x) and CΞDΛC is a loop originated from the descending boundary q(x), α and O are respectively the upper and lower closure points of the adsorption isotherm. The complexion diagrams for common points are shown too; hatched areas indicate the correction needed to be taken into account for the adsorbed film in order theorem-6 to be valid. Note that the thickness of the film increases with pressure.

**Fig 3:**
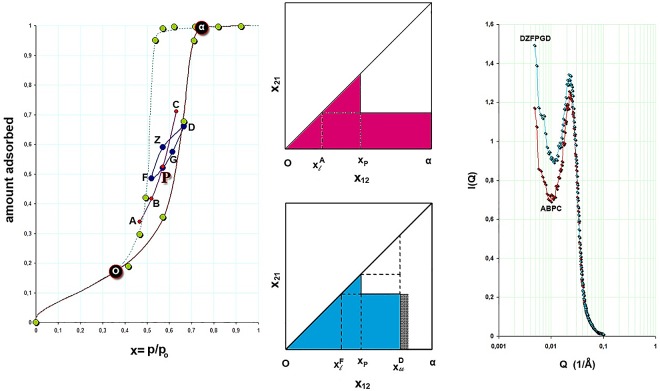
Scanning of the hysteresis loop *in situ* with SAXS. The CH_2_Br_2_ adsorption isotherm is normalized; outer solid line is for the adsorption branch and dash line for the desorption branch; green points correspond to the reconstruction of the adsorption isotherm from SAXS data. Point P is approached from two different paths; ΑBPC is a primary ascending curve originated from the desorption branch and FPGD is a secondary ascending curve of the DZFPGD loop originated from the adsorption branch. The complexion diagrams for P are shown and the hatched areas indicate the correction needed for the adsorbed film in order theorem-6 to be valid. The spectra which correspond to point P, where the aforementioned two different paths are converged, are shown too. At high-Q values the scattering curves coincide; this is not the case at low-Q values indicating different long-range correlations. The colors are consistent for easy inspection.

Figs 2 and 3 show the normalized CH_2_Br_2_ adsorption isotherm and the reconstruction of the adsorption isotherm from the SAXS data. The agreement with the actual isotherm is good (see Fig 3) if one takes into account the experimental errors, the sample differences, and the fact that many fewer points contribute to the reconstruction than the real isotherm.

## Results

### Effect of the Adsorbed Film

Fig 2 shows scanning curves of the CH_2_Br_2_ adsorption isotherm with common points between a primary descending curve AΛΞΟ and the loop CΞDΛC; the corresponding complexion diagrams are given too. Let us consider the effect of the adsorbed film on pore walls at points Ξ and Λ respectively. In the loop CΞ… the amount adsorbed at Ξ will be given by [44]:

$$V_{C\Xi }(x_{\Xi })=\iint_{\Sigma }\upsilon_{C\Xi }dx_{12}dx_{21}+\iint_{\Omega }w_{C\Xi }dx_{12}dx_{21}$$ (4)

where υdx_12_dx_21_ is the volume of the domains condensed with liquid (state Σ) and w is the amount adsorbed prior to capillary condensation (state Ω). Similarly, for the primary descending curve AΞ… the situation may be described as follows:

$$V_{A\Xi }(x_{\Xi })=\iint_{\Sigma }\upsilon_{A\Xi }dx_{12}dx_{21}+\iint_{\Omega }w_{A\Xi }dx_{12}dx_{21}$$ (5)

By subtracting V_CΞ_-V_AΞ_ at x_Ξ_:

$$0=\iint_{\Sigma }\left(\upsilon_{C\Xi }-\upsilon_{A\Xi }\right)dx_{12}dx_{21}-\iint_{\Omega }\left(w_{A\Xi }-w_{C\Xi }\right)dx_{12}dx_{21}$$ (6)

Since the complexion diagrams at Ξ indicate that υ_CΞ_-υ_AΞ_>0 it must also be Δw_Ξ_ = w_AΞ_-w_CΞ_>0; where Δw_Ξ_ is a correction for the adsorbed film that is needed in order Everett’s theorem-6 to be valid (hatched area). A similar situation may be described at point Λ. Likewise the inspection of the respective complexion diagrams shows that Δw_Λ_>Δw_Ξ_ (compare hatched areas) which is expected since the thickness of the film increases with pressure.

### Adsorption *In Situ* with SAXS

**Fig 4:**
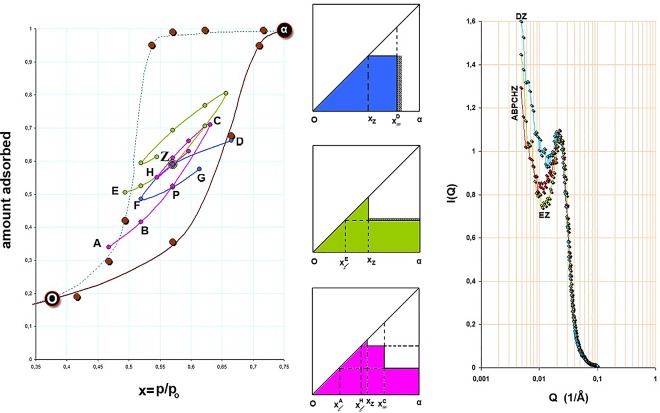
Scanning of the hysteresis loop *in situ* with SAXS. Point Z is approached from three different routes; ΑBPCHZ and EZ originated from the desorption branch of the adsorption isotherm and DZ originated from the adsorption branch of the adsorption isotherm. The complexion diagrams for Z are shown and the hatched areas indicate the correction for the adsorbed film. The spectra which correspond to point Z from the different routes are shown too. The colors and letters are consistent with Fig 3 for easy inspection.

Fig 3 shows a result of adsorption in conjunction with SAXS. The primary ascending curve ABPC meets the DZFPGD loop at point P (In Fig 4 –blue loop—the line GD is not drawn). The complexion diagrams show that apart of a correction for t-film (hatched area) the static macroscopic properties are similar. On the other hand the scattering curves although at high-Q values coincide, at low-Q values are different. This difference does not significantly contribute to the amount adsorbed (note that x-axis is logarithmic) but indicates a different order of the system in approaching point P.

Fig 4 shows a similar combined result as in Fig 3 but in this case the common point Z is approached by 3 different routes. The primary ascending curve EHZ meets the primary descending curve DZF at point Z; and the primary ascending curve ABPC turns at point C to a secondary descending curve CH and at H turns again to a secondary ascending curve that meets tangentially point Z. The complexion diagrams verify, with (including the) corrections for the t-film, similar macroscopic properties at point Z and the scattering curves verify the aforementioned differences at low-Q values.

We examined the scattering curves at various relative pressures by fitting the correlation peak with a normal distribution curve. Table 1 summarizes the result. The lower closure point (LCP; point O) corresponds to Kelvin radius r_k_ = 26 Å, Bragg spacing d = 251 Å and maximum relative intensity I_max_ = 2.1. For the sake of discussion the changes in d and I_max_ for the other relative pressures, p/p_o_, are normalized with these values. Plus/minus symbols indicate the direction of the change from previous p/p_o_. For Δ(Δd) a minus sign means that the system is influenced from smaller pores and for Δ(ΔI_max_) a plus means that the system holds less adsorbate (i.e. more empty pores).

**Table 1:** Scanning adsorption hysteresis *in situ* with SAXS.

CURVE	POINT		V_ads_ (%)	I_max_	p/p_o_	r_k_ (Å)	d (Å)	d (%)	I_max_ (%)	D_s_	D_m_	ξ_o_ (Å)
INITIAL	DRY		0	4.0	0		244			2.6		
	LCP	**O**	19	2.1	0.416	26	251	100	100	2.5		
BLUE LOOP	RPM	**D**	66	0.9	0.665	56	306	122	41	2.2	2.7	250
	COM	**Z**	59	1.1	0.571	41	296	118	51	2.4	2.5	250
	RPM	**F**	49	1.5	0.519	35	273	109	71			
	COM	**P**	52	1.3	0.571	41	277	110	62	2.3	2.6	150
		**G**	58	1.1	0.614	47	282	112	50			
RED	RPM	**A**	34	1.7	0.468	30	259	103	82	2.4	2.3	370
	COM	**P**	52	1.2	0.571	41	269	107	59	2.3	2.3	600
RED SUB-LOOP	RPM	**C**	71	0.1	0.631	50	299	119	4			
	RPM	**H**	55	1.2	0.545	38	286	114	55			
	COM	**Z**	59	1.1	0.571	41	286	114	51	2.2	2.4	250
GREEN	RPM	**E**	54	1.2	0.519	35	267	107	58	2.4	2.5	300
	COM	**Z**	60	1.1	0.571	41	273	109	50	2.1	2.6	250

**Fig 5:**
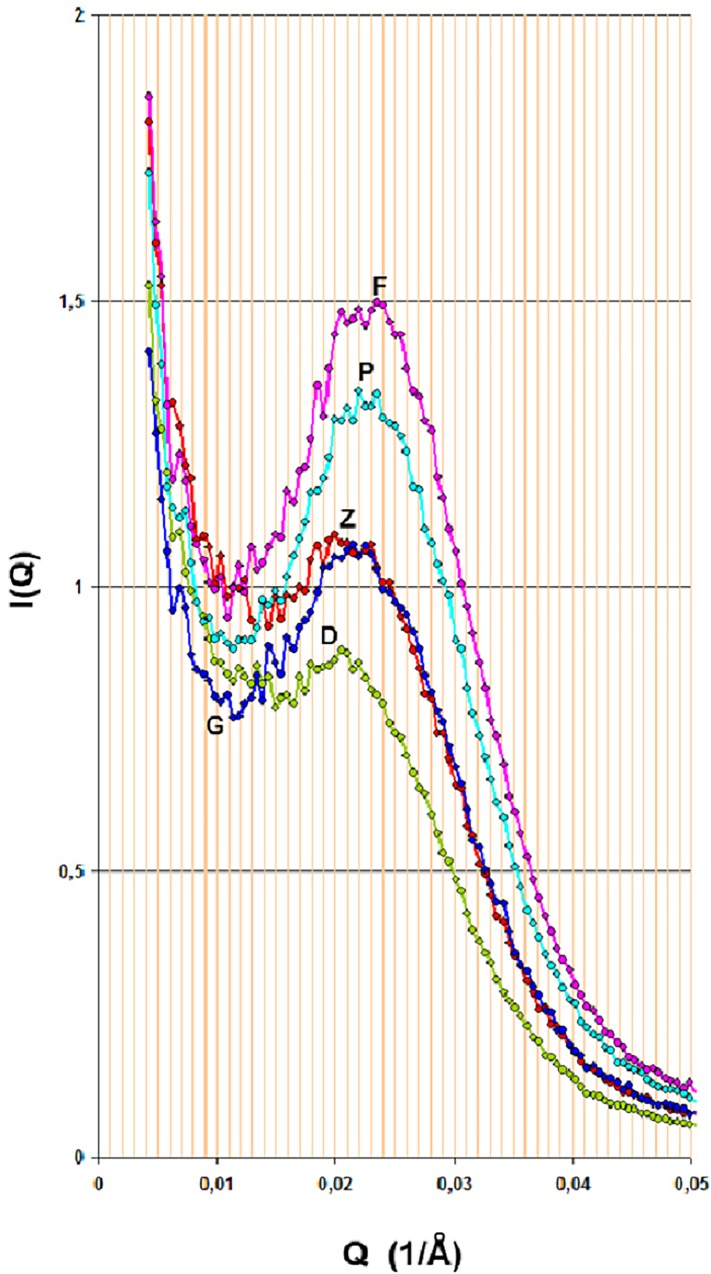
Scattering curves at various relative pressures around the loop DZFPGD; for full inspection and corresponding letters, view this figure in conjunction with Fig 3. Green: p/p_o_ = 0.665 point D; red: p/p_o_ = 0.571 point Z; pink: p/p_o_ = 0.519 point F; cyan: p/p_o_ = 0.571 point P; blue: p/p_o_ = 0.614 point G. Note that points Z and G although they correspond to almost equal amounts adsorbed their scattering curves red and blue, respectively, have different low-Q distributions indicating different metastabilities.

On the ascending boundary (see Fig 3 in conjunction with Fig 5), at p/p_o_ = 0.665 (point D) the scattered intensity is produced from pores greater than r_k_ = 56 Å. Bragg spacing is now d = 306 Å, Δd = 122% and ΔI_max_ = 41%; i.e. the scattering curve is influenced by larger pores whose population is not very large however. After a turn at p/p_o_ = 0.665 this point becomes an RPM and on the primary descending curve, at p/p_o_ = 0.571 (point Z), r_k_ = 41 Å and d = 296 Å. Pores greater than 41 Å are now empty. Although some new scatterers have been added into the system, hence Δ(ΔI_max_) = +10% relative to the previous p/p_o_ = 0.665, the rather small change in Δ(Δd) = -4% indicates that the scattering curve is still influenced by larger pores.

As desorption progresses, at p/p_o_ = 0.519 (point F), r_k_ = 35Å and d = 273Å, with Δd = 109% and ΔI_max_ = 71%; i.e. changes of -9% and +20% respectively from previous p/p_o_ = 0.571. At this point a rather major class of pores empties and the scattering curve is now influenced by smaller pores. After a turn at p/p_o_ = 0.519 this point also becomes an RPM and on the secondary ascending curve, at p/p_o_ = 0.571 (point P), only small changes in Δ(Δd) = +1% and Δ(ΔI_max_) = -9% are observed, relative to those corresponding to p/p_o_ = 0.519; i.e. the scattering curve is influenced by smaller pores. At p/p_o_ = 0.614 (point G), r_k_ = 47 Å and d = 282 Å. Although some pores have ceased to act as scatterers, hence Δ(ΔI_max_) = -12% relative to the previous p/p_o_ = 0.571, the small change in Δ(Δd) = +2% indicates that the scattering curve is still influenced by smaller pores.

In the loop DZFPGD the pores have been drained and refilled in a different order. Since this is a partially filled situation, pore blocking effects are not active at the moment and thus larger pores empty first. During pore refilling an adsorbed film thickens first. As it was shown in the previous paragraph the spectra corresponding to pore draining are influenced by larger pores whereas on refilling they are influenced by smaller ones, hence the correlation peak is located to lower Qs in the first and to higher Qs in the second case. This can be seen by comparing the scattering curves at points Z and G. At these points the system holds about the same amount of adsorbate yet the scattering curves differ at low-Q values although they coincide at high-Q values (Fig 5).

Now, at point A (p/p_o_ = 0.468) on the descending boundary pores with r_k_≥30Å are open. As a journey starts within the hysteresis loop this initial point becomes an RPM and a primary ascending curve (ABPC) is traced; i.e. refilling is influenced by smaller pores. At p/p_o_ = 0.571, Δd = 107% and ΔI_max_ = 59%. There is a small change in Δ(Δd) = +4% from the initial p/p_o_, and a large change in Δ(ΔI_max_) = -23%. The result indicates that although a number of pores has been condensed and thus ceased to act as scatterers, the large drop in I_max_, the spectrum is still influenced by smaller pores, hence the slight change in d. Prior to pore refilling an adsorbed film thickens on the pore walls and thus r_k_ becomes smaller in larger pores.

Since route (ABPC) converges with the loop (DZFPGD) at P the situation may be described as follows. At point P the system arrives from two different paths. The loop starts from an RPM that is influenced by larger pores whereas the route starts from an RPM that is influenced by smaller pores. It comes that the loop is free from pore blocking effects whereas the route is not. As a result d_loop_>d_route_ and this difference is consistently developed at low-Q values where the two spectra are different. Similar considerations may also be shown for point Z where 3 different paths are converged. When r_k_ at a given RPM is greater than another, then the Bragg spacing d at a common point that is originated from the path of the given RPM is greater than the other. It should also be mentioned that the d spacings presented at Table 1 are obtained after fitting the correlation peaks with a normal distribution.

### Mass Fractals

The low-Q part of the scattering curves is fitted with the structure factor S(Q) of mass fractals [56–58] with a dimension D_m_:

S(Q)∝ξoDmΓ(Dm)×sin[Dmarctan(Qξo)]Q[1+(Qξo)2]Dm2+const. (7)

where ξ_o_ is a characteristic size for the mass fractal.

**Fig 6:**
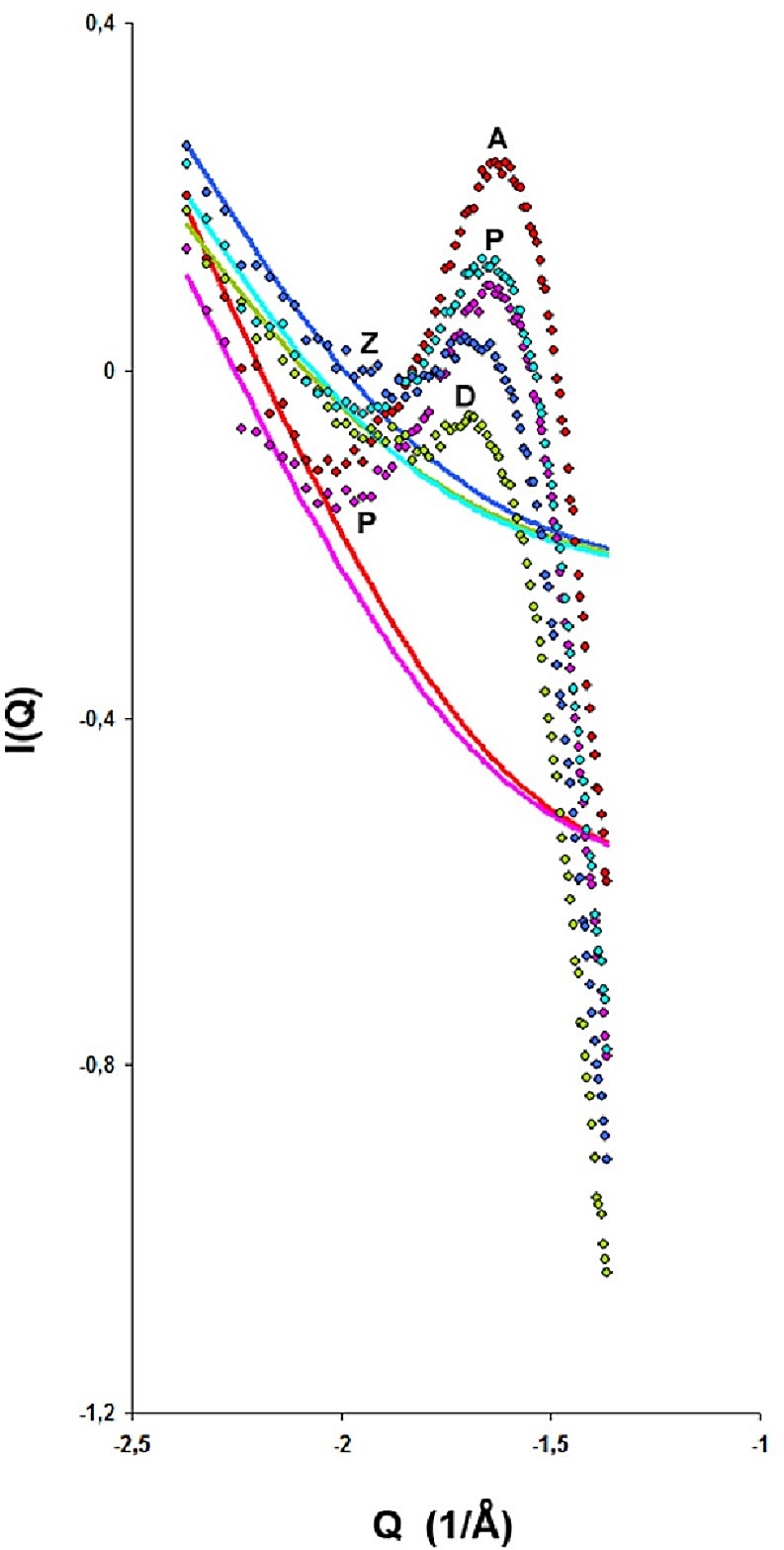
Mass fractals for the two different routes ABPC and DZFPGD of Fig 3. Green: p/p_o_ = 0.665 point D; blue: p/p_o_ = 0.571 point Z; cyan: p/p_o_ = 0.571 point P from path corresponding to loop; red: p/p_o_ = 0.468 point A; and pink: p/p_o_ = 0.571 point P from path corresponding to ABPC route. Note that that the mass fractal models of route ABPC (red and pink lines) correspond to larger ξ_ο_ than those of the loop (green, cyan and blue lines). The former route is originated from the desorption branch whereas the latter from the adsorption branch of the adsorption isotherm, hence the long-range correlations are more pronounced for the former than for the latter route.

**Fig 7:**
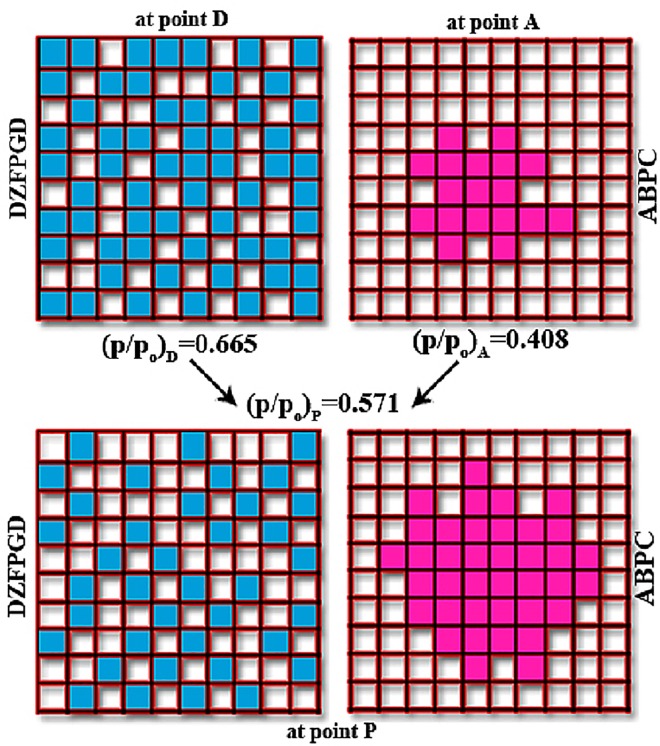
A sketch of different routes to common point P holding different distribution of equal amount adsorbed; letters and relative pressures are consistent with Fig 3.

Fig 6 shows the result; (ξ_o_)_loop_<(ξ_o_)_route_ indicating different long-range correlations for different RPMs and Table 1 records D_m_ and ξ_ο_ for the fits together with the surface fractal dimension which in the case of dry sample is D_s_ = 2.6. The large difference of ξ_ο_ for point P originated in the loop and point P originated from the route is due to the fact that desorption is driven by metastabilities discussed elsewhere [59]. Fig 7 shows schematically these long-range correlations; the drawing is based on an original sketch of Everett for co-operative effects [60]. During adsorption the domains are filled by following the Kelvin equation; that is, the smaller pores are condensed first. However, this pore blocking does not influence the adsorption process at all. Therefore domains may be filled in random (Fig 7 at point D). During desorption the picture is different because larger pores are blocked by smaller ones and they do not drain until the smaller ones empty first. Furthermore the pore network may exhibit co-operative effects (Fig 7 at point A).

Therefore domains may drain in large groups showing long-range correlations between them. Hysteresis effect, especially in the case of Vycor where the descending boundary is much steeper than the ascending one, may influence the desorption process with pronounced metastabilities [61]. In other words, for an RPM e.g. in the middle of the ascending boundary the domains are behaving more independently than an RPM on the descending boundary. Therefore entering the hysteresis loop from different RPMs different histories will be implanted to the paths toward the common point (Fig 7 at point P).

### Complementary Paths

The distribution of state Σ at point P was examined by subtracting the respective spectra from those corresponding to their RPMs, i.e.:

$${\left[\text{I}(\text{Q})\right]}_{\text{P}}^{\Sigma }={\left[\text{I}(\text{Q})\right]}_{\text{RPM}}^{\Omega }\;\text{-}\;{\left[\text{I}(\text{Q})\right]}_{\text{P}}^{\Omega }$$ (8)

**Fig 8:**
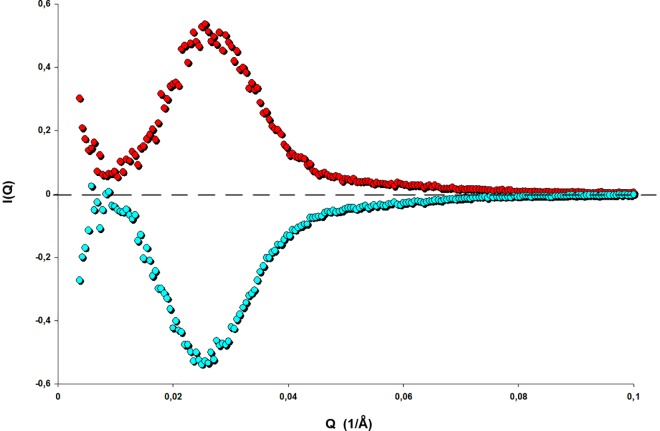
Complementary paths view in conjunction with Fig 3. Upper curve (red): Subtraction of the scattering curve at point P from the path ABPC at p/p_o_ = 0.571 from the scattering curve at point A at p/p_o_ = 0.468 of the same path. Lower curve (cyan): Subtraction of the scattering curve at point P from the loop DZFPGD at p/p_o_ = 0.571 from the scattering curve at point D at p/p_o_ = 0.665 of the same path. Note that the two curves are symmetrical except at low-Q values indicating that the paths AP and PD are complementary to each other.

The result shows that the two scattering curves are symmetrical to each other (Fig 8). By inspecting the scanning paths from A to P and the complementary one from P to D (see Fig 3) it can be seen that they are equal. Therefore for a point within the hysteresis loop having equidistant from boundary curves a prediction of the spectrum corresponding to a boundary point can be obtained if the other spectra are known.

## Discussion

According to Theorem-6 [33]:

1. Any point P within the hysteresis loop can be reached in an infinite number of ways, some from lower values of x, some from higher. The system will have definite static macroscopic properties at this point, but its state will not be completely defined since its behaviour when it moves away from P depends on the route by which this point was approached.
2. The paths to a given point P can be grouped into sets, or families, of paths so that all paths of a given set lead to the same domain complexion at P. Each set is called a set of cognate paths, and all paths in each set are characterized by the same major set of upper and lower bounds.

**Fig 9:**
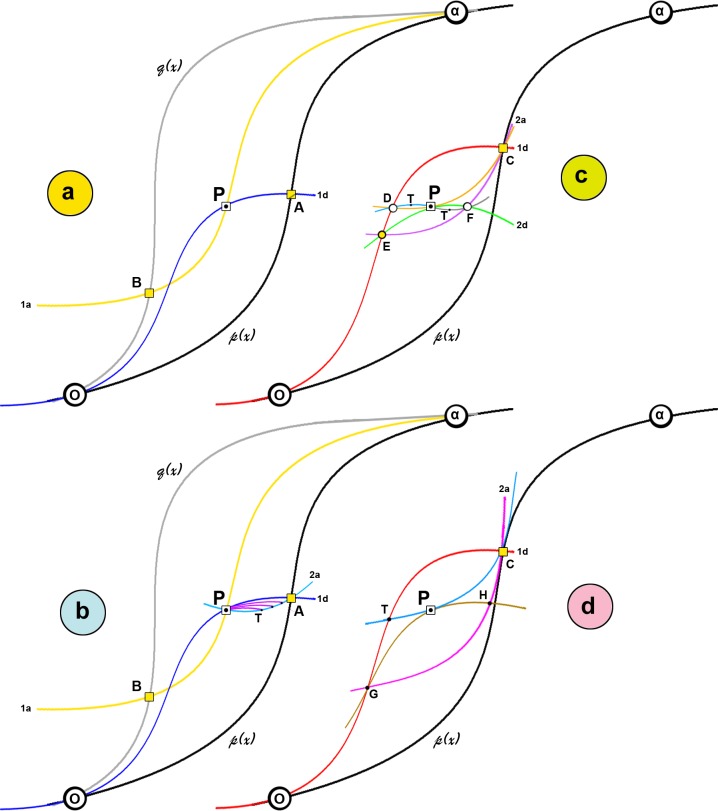
Different routes to a common point P; p(x) and q(x) are respectively the ascending and descending boundaries (i.e. adsorption/desorption branches); 1a and 1d denote primary ascending and descending curves; 2a and 2d denote secondary ascending and descending curves. Cognate paths are indicated and temporary points are denoted with T or T΄. In (c) and (d) the descending boundary has been omitted. See text for more details.

It is not clear why there is an infinite number of ways to reach point P. Fig 9 illustrates some different routes to a common point P. The system is composed from two strong memory points O and α which cannot be erased; that is the upper and lower closure points (U/LCP) of the hysteresis loop. Any point within this area can be directly approached by either a primary descending or a primary ascending curve (Fig 9A). To achieve this, the system has to make a turn at e.g. the ascending boundary. If one assumes that the appropriate turn is at point A, where a primary descending curve AO passes from point P; an RPM at point A (A_RPM_) is thus created. Therefore at point P the system has one RPM.

In this case the pressure x can be either decreased or increased. By decreasing the pressure the system will move to the LCP and erase A_RPM_. By increasing the pressure the system will move to A via the route PTA (Fig 9B). Note that point P is now an RPM. Again at the point A one can either increase the pressure towards the UCP and thus erase RPMs or can enter the hysteresis area through the same path AO. However the system on the way from P to A may take a turn back to P. At the turning point another RPM is created and now the system has 3 RPMs. When the system reaches at P the last turning point will be erased and P will cease to act as an RMP; on the way back to A another turning point may be constructed and then erased and so on creating thus cognate paths of temporary RPMs from a family of secondary descending curves (Fig 9B, purple lines).

Another, indirect this time, route to approach point P is from a higher primary descending curve (e.g. CO, Fig 9C); primary descending curves lower than AO cannot reach at P. Let’s assume that D is a turn point on curve CO that passes through P. Therefore at P the system has two RPMs (C_RPM_ and D_RPM_). Now it can be moved either to point C or back to point D via the path PTD by creating a family of tertiary ascending curves around the PTDP loop and corresponding temporary RPMs in this cognate path that will eventually collapse when the system moves away. Another route to approach P is the CEF and at F a turn to P. At P the system has three RPMs (C_RPM_, E_RPM_ and F_RPM_). A cognate path from a family of tertiary descending curves in the loop PTFP can now be created.

More routes to point P are possible but not infinite. For example GHP or TP (Fig 9D) are not valid paths because they violate theorem 3. Notice that the slopes of routes GHC and TPC are sharper than the slope of the ascending boundary p(x). The same limitations also apply to higher primary descending curves than e.g. CO, etc. Similar considerations can be made for routes initiated from the descending boundary. In an early formulation of Theorem 6 Everett suggested that point P within the hysteresis loop can be reached in a number of ways. Although cognate paths are not included in this formulation it is more rigorous than the final version of Theorem-6 where an infinite number of ways is claimed.

Going back to Fig 3 it is interesting to review why a system approaching point P from e.g. the route AP does not exit the hysteresis loop by turning to PD but it continuous its journey on the original APC route. At a common point the same average macroscopic state can be built up from different microscopic states; the complexion diagrams in the same figure shows this difference. As a result at P a system from one path is not possible to exit the loop by changing its path because different routes hold different metastable states sustained by hysteresis effects. As a matter of fact these metastabilities are highlighting the way of a system back to an RPM.

Although the concept of RPM is more appropriate for relaxation phenomena rather than hysteresis, it was found to be a characteristic feature of capillary condensation systems too. To this end Middleton’s no-passing rule can be modified accordingly. Let p(x) be the ascending boundary curve which is evolved under a chemical potential μ^p^(x) and similarly q(x) is a primary descending curve evolved under μ^q^(x). By entering the hysteresis loop from a point p/p_o_ = x_o_, p(x_o_)≥q(x_o_) and μ^p^(x)≥μ^q^(x). Then it will remain true that p(x)≥q(x) for all p/p_o_. As a matter of fact it is always p(x)>q(x) and only in the limiting case where x = x_o_ p(x) = q(x). This can be easily seen by turning the isotherm 90° counter clock wise. Now if a system q(x_o_) is evolved under μ^q^(x), where μ^q^(x)<μ^q^(x_o_) for x<x_o_ the final state of the system depends only on μ^q^(x_o_). In particular, a system coming back to a previous extremal field will return to exactly the same point x_o_ because q(x) cannot exceed p(x) due to no-passing rule and again domains which are empty will fill over a small range of x in a relative rapid way of equilibration; i.e. q(x) will return to x_o_ in an escalating way which is equivalent to the adiabaticity assumption. That is x_o_ is an RPM.

## Conclusions

Theorem-6 of the domain theory was examined by conducting adsorption *in situ* with SAXS. At a common point the system can reach in a finite number of ways, some from lower and some from higher relative pressures.

The results indicate that a correction for the thickness of the adsorbed film prior to capillary condensation is required in order the classical theory to be satisfied. The complexion diagrams are constructed and then the necessary corrections have been added. At a common point, systems from different routes although they have similar macroscopic properties, they differ in microscopic states; that is, they hold different metastabilities. For instance the chemical potential of a small nucleus of state Σ in Ω will be greater than that of bulk Σ. These metastabilities are sustained by hysteresis effect and the steepness of the adsorption isotherm is a measure of the system's ability to contain states of widely different saturation coexisting at equilibrium in neighbouring regions of the pore space at the same p/p_o_.

At the common point the spectra also vary at low-Q scattering intensities indicating different long-range correlations. Mass fractal analysis of this region verifies these differences. On the other hand, because the scattering curves coincide at high-Q values, when two different paths are initiated from different points on the boundary curves and they are equidistant from the common point their scattering curves will be complementary at the high-Q regime.

The evolution of a system within the hysteresis loop is influenced by the RPM from which is originated. Although the memory points in general refer to relaxation phenomena they also constitute a characteristic feature of capillary condensation. We have discussed the analogies of the no-passing rule and the adiabaticity assumption in the frame of hysteresis.

The scattering curves at various points of a scanning loop (including two common points) were compared to each other. Within the loop the pores drain and refill in a different order. As a result the loop is open. Domain drainage does not seem to leave much of an adsorbed film behind it; only possible metastabilities of state Σ in Ω. On the other hand domain refilling requires an adsorbed film to thicken on the pore walls prior to capillary condensation. The scattering curves at points Z (drainage) and G (refilling) demonstrate this critical difference. The spinodal peak corresponding to point Z is broader and locally skewed whereas that corresponding to point G is sharper; i.e. in the former case the situation reflects a wider pore size distribution than in the latter one. Prior to pore refilling an adsorbed film thickens on the pore walls and thus r_k_ becomes smaller in larger pores. Laplace metastable surfaces of constant curvature have already been discussed in the literature [62]. Hysteresis effects can maintain such a situation as frozen-in metastability. In the case of unduloids the Kelvin equation can be modified within the hysteresis loop as:

$$\ln\frac{p}{p_{o}}=-\frac{\gamma V_{L}}{RT\alpha }$$ (9)

where α is related to the mean curvature of unduloid configuration C = -1/α with 1/α = (1+e)/R_c_ where R_c_ is the radius of the cylindrical domain capillary and e is the rolling ellipse eccentricity taking values 0≤e≤1. It is noted, however, that some values of (1+e) may represent unstable configurations.
